# Goblet Cells and Mucins: Role in Innate Defense in Enteric Infections

**DOI:** 10.3390/pathogens2010055

**Published:** 2013-02-04

**Authors:** Janice J. Kim, Waliul I. Khan

**Affiliations:** 1 Farncombe Family Digestive Health Research Institute, McMaster University, 1280 Main St W, Hamilton, Ontario, L8S 4K1, Canada; E-Mail: janice.kim@mcmaster.ca (J.J.K.); 2 Department of Pathology and Molecular Medicine, McMaster University, 1200 Main St W, Hamilton, Ontario, L8N 3Z5, Canada

**Keywords:** Goblet cells, mucins, enteric infection, innate defense

## Abstract

Goblet cells reside throughout the gastrointestinal (GI) tract and are responsible for the production and preservation of a protective mucus blanket by synthesizing and secreting high molecular weight glycoproteins known as mucins. The concept of the mucus layer functioning as a dynamic protective barrier is suggested by studies showing changes in mucins in inflammatory conditions of the GI tract, by the altered goblet cell response in germ-free animals, and by the enhanced mucus secretion seen in response to infections. The mucin-containing mucus layer coating the GI epithelium is the front line of innate host defense. Mucins are likely to be the first molecules that invading pathogens interact with at the cell surface and thus, can limit binding to other glycoproteins and neutralize the pathogen. This review will focus on what is known about goblet cell response in various GI infections and the regulatory networks that mediate goblet cell function and mucin production in response to intestinal insults. In addition, we describe the current knowledge on the role of mucins in intestinal innate defense. It is the aim of this review to provide the readers with an update on goblet cell biology and current understanding on the role of mucins in host defense in enteric infections.

## 1. Introduction

The luminal side of the mucosal layer of the gastrointestinal (GI) tract is covered by a mucus blanket which provides protection to the mucosa from dehydration and mechanical damage and constitutes a physical barrier between the underlying epithelium and luminal contents, which include pathogenic bacteria, viruses, and parasites [1,2]. Nevertheless, mucus is permeable to low molecular weight components and this property is important for the intestinal absorption of nutrients. Mucus contains as its major components large molecular weight glycoproteins known as mucins secreted by goblet cells [3,4]. Goblet cells are present throughout the GI tract and are the main source of mucins in the intestinal tract [5]. The concept of the mucus layer functioning as a dynamic protective barrier is suggested by studies showing changes in mucins in inflammatory conditions of the gut, by the altered goblet cell response in germ-free animals, and by the enhanced mucus secretion observed in response to infections [6,7]. 

The GI tract is constantly exposed to large numbers of food and water borne pathogens, as well as an endogenous microbiota adapted to symbiotic living within the gut. Despite this challenge, however, the GI tract is rarely overwhelmed by microbial pathogens, suggesting that it has effective innate and systemic defense systems. Goblet cells, epithelial cells, macrophages, and dendritic cells are the major cellular constituents of the innate defense system, and the mucus layer containing mucins represents the front line of this system [5,8]. T and B cells are the major combatants of the adaptive immune system which builds the final defense line both as master regulators and as an inducible system to remove pathogens that have circumvented innate defense lines. An association with an alteration of goblet cell response and mucin production is observed in various enteric infections caused by bacteria, viruses, and parasites [2,9,10]. In addition, quantitative and qualitative alterations in mucins are observed in other GI diseases such as ulcerative colitis [11], colon carcinoma [12] and coeliac disease [13]. Taken together, there is now abundant evidence to suggest an important role of goblet cells and mucins in various GI disorders, necessitating precise understanding of goblet cell biology and function of mucins in pathology, pathophysiology, and host defense within the GI tract.

## 2. Goblet Cells: Origin and Differentiation

In the GI tract, goblet cells, as well as the three other principal cells (enterocytes, enteroendocrine cells and Paneth cells) arise from a multipotent stem cell located near the base of the crypts of Lieberkühn [14]. Current thinking is that progenitor cells, located in the proliferative compartment, derive from these stem cells and while leaving the crypts, further differentiate into each of the intestinal cell lineages [15,16]. Goblet cells appear early in development (in the human fetal small intestine, at 9–10 weeks’ gestation) and their morphology is shaped by the distended theca containing mucin granules located below the apical membrane [17]. Kinetic analysis of goblet cell dynamics in mouse intestine shows that once propagated, goblet cells migrate from the base of the crypt to the villus tip, where they are sloughed into the lumen [18]. This progression from birth to death occurs in mice within 2–3 days; thus, the population of goblet cells is short lived and is constantly undergoing replacement. Goblet cells undergo dramatic morphological changes during their life span. As evidenced in morphometric studies in rabbit colon [19], once propagated from stem cells at the base of the crypt, immature goblet cells begin to rapidly synthesize and secrete mucin granules. These immature goblet cells at the crypt base are large and pyramidal in shape and as the cell progresses toward the colonic surface, it diminishes in volume, shedding cytoplasm and organelles trapped between mucin granules as the granules are secreted. During this volume reduction, cell morphology changes; contact with the lumen increases, and organelles become segregated. At the mouth of the crypt, the cell obtains its characteristic cup-like shape; the apical portion of the cell is distended and packed with mucin granules, and the basal portion of the cell is narrowed into the “stem” of the goblet in which the nucleus and synthetic organelles reside. Although goblet cells are distributed through the entire length of the mammalian GI tract, their contribution to the total epithelial volume is not constant. In the rat small intestine, the volume density of goblet cells increases aborally, from the duodenum to distal ileum [5]. This trend continues in the large intestine with the density of goblet cells in the colonic epithelium also increasing proximal to distal, from cecum to rectum. The maintenance of stem cells and differentiation into specific cell lineages in the intestine involves a complex interplay of multiple developmental pathways including Wnt/β-catenin, bone morphogenic protein (BMP), and PI3-kinase/Akt signaling [20]. Basic helix-loop-helix (bHLH) gene Math1 is a mammalian homolog of the *Drosophila atonal* gene and is expressed in the GI tract during development and has been identified in both the immature crypts and villi of the intestinal epithelium [21]. Math1 appears to be important for specification of intestinal secretory lineages as Math1 deficient mice fail to develop three of the four GI mucosal cell types—goblet, Paneth, and enteroendocrine cells.

## 3. Intestinal Mucus and Mucins

Intestinal mucus is a highly hydrated aggregate that serves as the frontline of innate host defense against endogenous and exogenous irritants, microbial attachment and invasion, but allows for the transport of nutrients. Mucins act as the main structural component of the intestinal mucus layer and are secreted mainly by goblet cells that are dispersed throughout the epithelial layer. Mucins are the key polymeric, viscoelastic and protective components of mucus. In addition to mucins, mucus is comprised of water, ions, and molecules of the immune systems such as immunoglobulin A (IgA) and anti-microbial peptides, which facilitate the clearance of pathogenic organisms [22,23,24,25,26]. 

Mucins are large glycoproteins with an average molecular mass of 2 × 10^6 ^Daltons [27] that assemble into a protective gel-like layer that extends upwards to 150 μm of the epithelial surface [28]. Mucin glycoproteins consist of linear or branched oligosaccharide chains attached to a protein core containing a very distinct composition of amino acids. Serine (Ser) and threonine (Thr) are even more prevalent in core proteins, making up 41% of the molecule in humans [29] and 57% in rats [30]. Both Ser and Thr have side chains that contain hydroxyl groups that are involved in linking oligosaccharide chains to the protein *viaO*-glycosidic bonds [31]. Core proteins also contain a high percentage of proline (Pro), aspartic acid (Asp) and glutamic acid (Glu). The oligosaccharide side chains are formed by five sugars: fucose (Fu), galactose (Gal), *N*-acetylgalactosamine (GalNAc), *N*-acetylglucosamine (GlcNAc), and sialic acid (SA). These sugars form linear or branched arrays of 2–12 sugar residues that are attached *via* an *O*-glycosidic bond between Ser or Thr in the protein and GalNAc. 

## 4. Goblet Cells and Mucins in Intestinal Infections

### 4.1. Parasitic Infections

Changes in goblet cell response and mucin production are observed in many intestinal infections caused by parasites, bacteria, and viruses. Hyperplasia of mucin-secreting goblet cells has been described in a number of helminth parasitic infections including *Ascaris galli* [32], *Trichinella spiralis* [33,34], *Nippostrongylus brasilensis* [2,35,36], and *Trichuris muris* [37,38]. In addition to an increase in goblet cell numbers, qualitative changes in the composition of mucins in goblet cells are observed during infection with *N. brasiliensis*. Here, the composition of intestinal goblet cells changes from containing neutral to acidic (sulphated) mucins and alteration of terminal sugars of goblet cell mucins has been observed around the time of worm expulsion [39,40]. Putative mechanisms underlying the protective role of mucins against parasites include the demonstration of trapping of worms in the mucus and inhibition of parasite motility and feeding capacity [2,41]. In addition to enhancing the mucus barrier, goblet cells may play a role in immune activation by presenting luminal antigens to lamina propria dendritic cells [42]. 

Mucin genes Muc2 and Muc3 have been shown to be up-regulated in *T. spiralis* infection [43]. Increased amounts of mucins and alterations in Muc2 have also been reported in rats infected with *N. brasiliensis* [44,45]. In a recent study, we have shown that Muc2 mucin is an important component of innate defense in nematode infection by utilizing resistant, susceptible, and Muc2-deficient mice [37]. We observed an increase in Muc2 mucin exclusively in resistant mice correlating with worm expulsion following *T. muris* infection. In addition, the mucus barrier in resistant mice was less permeable than that of susceptible mice [37]. Interestingly, an increase in Muc5ac, a mucin normally expressed in the airways and stomach, was observed in the colon following *T. muris* infection of only the resistant strains. Around the time of expulsion, there was a remarkable increase in Muc5ac expression in Muc2 deficient mice triggered by the infection [37]. In a subsequent study, Muc5ac was shown to be critical in the expulsion of *T. muris* in mice whereby loss of Muc5ac resulted in susceptibility to chronic infection [38]. In addition, Muc5a affects the overall porosity of the mucin network within the GI tract, creating a niche that may affect worm viability [38].

Among the protozoan parasites, the *Entamoeba histolytica* model is extensively used for gaining insight into host-parasite interactions in the context of host mucins. Colonic mucins form a barrier that protects the host and inhibits amoebic adherence to the underlying epithelial cells. In the *E. histolytica*-colitis model, it is shown that the luminal mucus barrier and goblet cell mucin stores are depleted prior to amoeba contact with and invasion of the underlying mucosa [46]. Although the cause of the mucin depletion is unknown, it is reasoned that *E. histolytica*-derived secretagogues and mucinase activity may deplete mucin stores as a means of evading the mucus barrier [47]. *E. histolytica* can invade through the mucus layers by secreting cysteine proteases, which cleave the MUC2 mucin, resulting in a defective mucus barrier through which it can invade and attach to the intestinal mucosal cell surface [48]. Gal and GalNAc sugar residues of purified colonic mucins competitively inhibit amoeba binding to host epithelium by creating a physical barrier between the parasite and the epithelium [49]. In an *in vitro* model, it has been shown that that both crude and purified colonic mucins derived from LS 174T cells prevent amoebic adherence to Chinese hamster ovary cells [50]. *E. histolytica* can bind to mucins, which is necessary for colonization; it may also degrade mucins to ease entry into the underlying epithelium, which causes disease. It is shown that cysteine proteases secreted from *E. histolytica* disrupts the mucin polymer network and consequently aids in breaking down the protective mucus barrier [51]. Among other intestinal protozoan parasites, *Giardia lamblia*, *Tritrichomonas suis*,and *Tritrichomonas mobilensis* are capable of producing a variety of mucin-degrading enzymes which may participate in degrading host mucins and hence aid in the penetration of the host mucus barrier [52,53,54].

### 4.2. Bacterial Infections

Several GI bacteria have developed specific pathogenic factors and/or ways of interfering with mucin production in order to enable them to cross the mucus barrier. *Helicobacter pylori* is one such bacteria and colonizes the gastric mucus gel layer by means of a very close association with MUC5AC mucin and MUC1 [55]. The MUC5AC glycoprotein is the primary receptor for *H. pylori* in the human stomach [56]. Altered expression and allelic association of the hyper-variable membrane mucin MUC1 is observed in *H. pylori* gastritis [57]. *H. pylori* uses its flagella for motility within the gastric mucus layer [58]. In addition, *H. pylori* reduces mucin exocytosis [59], decreases gastric mucin synthesis [60], and causes an abnormal expression of the gastric mucins MUC1, MUC5AC, and MUC6 [61]. Recent studies suggest modulation of virulence factor adhesin BabA expression during colonization of *H. pylori* [62]. Adhesin BabA binds the blood group antigen Lewis b (Leb) and related antigens [63]. As mucins of the stomach also carry Leb antigens, modulation of BabA expression during the infection may contribute to the penetration of mucus barrier and colonization of the bacteria [64]. It is also suggested that increased mucus viscosity in patients with gastroduodenal disease decrease *H. pylori* motility *in vivo* [65]. The inflammation-associated mucin sialylation has been observed to return to the normal pattern following successful bacterial clearance with antibiotic use in patients with *H. pylori* infection [66,67]. 

In *Salmonella* infection, interferon (IFN)-γ receptor-signaling influences the generation of mucus-filled vacuoles by goblet cells and mucus release into the gut lumen [68]. Utilizing the HT29 Cl.16E human tissue culture model, it has been shown that Cholera toxin stimulates mucus secretion [69]. In *Citrobacter rodentium* infection [mouse model of Enteropathogenic *E. coli* (EPEC) and Enterohemorrhagic *E. coli* (EHEC)], it has been shown that Muc2 production plays a critical role in host protection by limiting overall pathogen and commensal numbers, suggesting that Muc2-dependent mucus production is critical for effective management of both pathogenic and non-pathogenic bacteria during infection by an EPEC/EHEC-like pathogen [70]. Secreted mucus has already been reported to act as a barrier to enteroinvasive *Yersinia enterocolitica* [71] and *Shigella flexneri* [72].

### 4.3. Commensal Bacterial and Mucins

The GI tract is colonized by a complex, dynamic microbial ecosystem. The resident microbiota in the gut constitutes a heterogeneous microbial ecosystem containing up to 1 × 10^14 ^CFU of bacteria [73]. The normal intestinal mucosal epithelium has tolerance to commensal microbiota because of its ability to distinguish commensal microbiota from pathogenic microorganisms by their molecular patterns, such as microbe associated molecular patterns and pathogen-associated molecular patterns, through pattern recognition receptors (PRRs) such as cell surface Toll-like receptors (TLRs) and cytoplasmic nucleotide-binding oligomerization domain like receptors (NLRs) [74]. GI microbes can regulate mucin production by activating different signaling cascades and secretory elements. Commensal bacteria and probiotics have the ability to modulate the normal intestinal microbiome by direct action and also by signaling the host, which in turn adjusts the immune response [75,76]. Probiotics such as *Lactobacillus plantarium* were reported to induce Muc2 and Muc3 mucins and inhibit the adherence of EPEC (enteropathogenic *E. coli*), indicating that enhanced mucus layers and glycocalyx overlying the intestinal epithelium and the occupancy of the microbial binding sites by *Lactobacillus* spp. provide protection against invasion by the pathogens [77]. Probiotics also cause qualitative alterations in mucins, preventing pathogen binding. Bacterial products such as lipopolysaccharides (LPS) and flagellin A from Gram-negative bacteria and lipoteichoic acids (LTA) from Gram-positive bacteria are the most common modulators of mucin production by mainly affecting Muc2 and Muc5ac mucins [48]. In a recent study, it has been shown that successful establishment of the chronically infecting parasitic nematode *T. muris* in the intestine is dependent on intestinal microflora and corresponds with modulation of the host immune response [78]. These findings suggest that there are important interactions between intestinal microflora and parasites in generation of immune response and host protective immunity.

### 4.4. Viral Infection

Among viral infections, rotaviruses are the leading cause of severe viral gastroenteritis in young children. Up-regulation of Muc2 expression and alteration of mucin structure is observed in the course of infection in a murine model of rotavirus infection [79]. Mucins isolated from control and infected mice were shown to be able to efficiently neutralize rotavirus infection in vitro [79]. In addition, mucins isolated from infected mice were more potent in inhibiting rotavirus infection than mucins from control mice [79]. This suggests that goblet cells and mucins play a role in the host defense against rotavirus infection.

## 5. Regulation of Goblet Cell Response and Mucin Production

After synthesis in goblet cells, mucins are packaged into granules, transported to the cell surface and secreted into the lumen. Mucins are secreted by two pathways, constitutive or basal secretion, which is low-level continuous secretion dependent on cytoskeletal movement of secretory granules, or stimulated or regulated secretion, which involves exocytosis of granules in response to external stimuli such as neuropeptides, cytokines and lipids, respectively [48,80]. Mucin secretagogues are known to signal through various secondary messengers that include intracellular Ca^2+^, cAMP and diacylglycerol, which activates protein kinase C to stimulate mucin secretion [46,48,81]. While infection with various enteric infectious agents leads to alteration in goblet cell and mucin responses, the precise mechanisms regulating goblet cell biology and mucin production remain unknown. 

Goblet cell hyperplasia in many nematode infections has been suggested to be under immunological control [33,34,35]. In *N. brasiliensis* infection, it has been also shown that treatment of mice one day prior to infection with a single dose of anti-CD4 antibody significantly reduces the amount of intestinal mucus produced and inhibits the normal spontaneous expulsion of worms [36]. In addition, activation of mucosal T lymphocytes from duodenal biopsies with monoclonal anti-CD3 antibody significantly increases both radiolabelled glucosamine incorporation into glycoproteins and secretion of radiolabelled glycoproteins *in vitro* [82]. These observations clearly suggest an important role for T cells in infection-induced intestinal goblet cell hyperplasia and mucus production. Th2 type cells are important in host protective immunity to many intestinal nematode infections including: *T. spiralis*, *N. brasiliensis*, *and T. muris*, and are characterized by expression of cytokines such as interleukin (IL)-4, IL-5, IL-9, IL-10, and IL-13 [83,84,85]. Signal transducer and activator of transcription factor 6 (Stat6) is important in the generation of a Th2 response and studies from our laboratory have shown a critical role for Stat6 in the development of goblet cell hyperplasia during *T. spiralis* infection [86]. Stat6 deficient mice infected with *T. spiralis* failed to develop infection-induced goblet cell hyperplasia as compared to infected wild-type mice. In addition, we also observed a significant attenuation of *T. spiralis* infection-induced goblet cell hyperplasia in studies where the immune response was shifted from Th2 to Th1 as a result of an IL-12 gene transfer [87]. Recently, a role for IL-22 (produced by innate immune cells such as Th17 cells) in regulating early host defense against *C. Rodentium* infection has been identified, whereby IL-22 deficient mice had compromised epithelial integrity [88]. IL-22 receptor chains are expressed on colonic epithelial cells (including goblet cells), suggesting that IL-22 may directly target these cells to induce anti-microbial responses or protect the epithelial layer from bacterial invasion and damage [88]. In addition, IL-22 has been shown to stimulate mucus production (enhancing the production of mucins Muc3, 10, and 13) and goblet cell restitution under intestinal inflammatory conditions [89]. Taken together, these studies clearly suggest that Th2 cytokines are strong candidates for the proliferation/ differentiation of intestinal goblet cells during nematode parasite infection. In addition, a recent study has shown a role for IL-10 in preserving the intestinal mucus barrier and prevention of protein misfolding and ER stress in goblet cells [90].

Goblet cells are also the main source of intestinal trefoil factor (ITF). ITF is considered to interact with mucin to increase the viscosity of the mucus gel and one hallmark of the differentiation of intestinal goblet cells is the early expression of ITF [91]. Recently, it has been demonstrated that IL-4 and IL-13 up-regulate ITF from mucin producing HT-29 CL.16E and HT 29 cells through the Stat6 pathway [92]. In addition, it has also been shown that in human colon cancer cells, mRNA of MUC2 is stimulated by IL-4 and IL-13 through a mitogen-activated protein kinase pathway [92]. As IL-4 receptors have been shown to be present on intestinal epithelial cells, cultured airway epithelial cells and H-29 CL.19E cells [91,94], it is reasonable to presume that Th2 cytokines such as IL-4 and IL-13 play an important role in ITF and mucin production from intestinal goblet cells. 

## 6. Conclusions

Herein, we provide readers with an update on intestinal goblet cell biology and mucins in the context of innate defense in enteric infections. The studies described show that alteration in goblet cell function and mucin production takes place in a number of enteric infections. Table 1 summarizes goblet cell and mucin responses in several common parasitic, bacterial, and viral infections.

**Table 1:** Alteration in goblet cells and mucins responses during common enteric infections.

Enteric Infection		Goblet Cell & Mucin Response	Reference
*Parasitic Infection*	Helminth Infection		
	*N. brasiliensis*	Goblet cell hyperplasia; 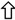 Muc2,	[39,40,44,45]
		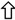 sulfated mucins	
	*T. spiralis*	Goblet cell hyperplasia; 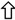 Muc2/3	[43]
	*T.muris*	Goblet cell hyperplasia; 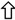 Muc2/4/13/17, 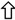 charged mucins	[37,38,95]
	Protozoal Infection		
	*E. histolytica*	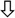 Mucins	[56]
*Bacterial Infection*	*C. Rodentium*	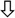 Mucins 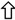 Muc1	[96]
	*H. pylori*	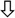 Muc6/5ac, 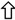 Muc4	[61,97,98]
	*C. jejuni*	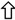 Muc1	[103]
*Viral Infection*	*Rota virus*	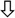 Goblet cell (early time points);	[79]
		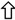 Muc2, 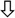 sulfated mucins	

**Figure 1:**
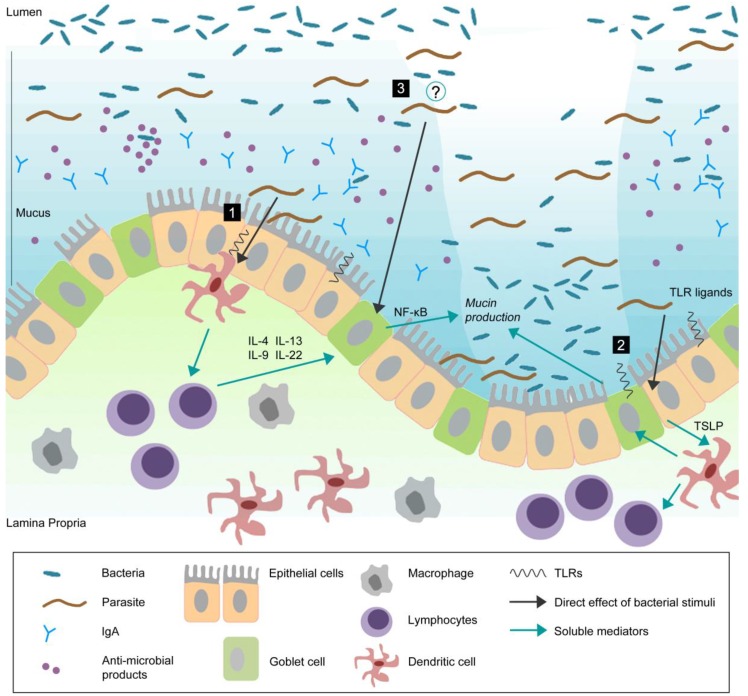
Goblet cells reside throughout the gastrointestinal (GI) tract and are responsible for the production and preservation of a protective mucus layer by synthesizing and secreting mucins.

During the last decade, substantial improvements in analytical techniques coupled with information of mucin genes have provided new insights into the biology of goblet cells, the role of mucins, and the regulatory networks that mediate mucin production in response to intestinal insults, including infections. A number of important notions have been generated from the studies on the interaction between enteric pathogens and mucins. First, enteric infections by parasites, bacteria, and viruses can influence goblet cell biology by acting on the differentiation pathway of stem cells towards goblet cells and by modulating the synthesis and release of mucins. According to the existing literature, models of Th2-based nematode infections provide the most comprehensive information on infection-induced goblet cell hyperplasia and increased mucin production. Among bacterial infections, many studies have looked at mucins in *H. pylori* infection and have revealed important information on bacteria-mucin interactions in the gastric mucosa. An emerging role for mucins has also documented in *Salmonella*, *Shigella*, and *Citrobacter* infections. Most of the information on the role of mucins in enteric viral infection comes from studies using rotavirus infection. Further studies on goblet cells and mucins in other viral infection models will help to enhance our knowledge on mucins in enteric viral infections. Another important notion that has emerged is that the immune system plays an important role in the regulation of intestinal goblet cell hyperplasia and mucin production. The data discussed in this review strongly suggest that the immune system, particularly the adaptive component, plays an important role in the development of nematode infection-induced intestinal goblet cell response, and that this immune-mediated change in intestinal physiology is associated with host defense. Increased mucus may trap the parasite, prevent attachment to the epithelial surface, inhibit parasite motility and feeding capacity, and subsequently, an enhancement in propulsive activity assists the expulsion of the parasites from the gut. Another important emerging concept is the interaction between commensal bacteria and goblet cell response and mucin production. It would be important and interesting to investigate whether enteric pathogens can modulate goblet cell biology and mucin production by changing the composition of gut flora and subsequently influencing the interaction between gut flora and goblet cells (Figure 1).

This thick mucus layer blankets the intestinal epithelium. Commensal bacteria remain in the outer mucus layer while the inner layer remains resistant to penetration by these microbes. Epithelial cells further strengthen this barrier by secreting anti-microbial products such as lysozymes and α-defensins that help to eliminate bacteria that threaten to cross the mucus barrier [99]. Secretory antibodies, IgA (produced by B cells in the lamina propria), are secreted by epithelial cells into the mucus and further prevent bacteria from penetrating into host tissue [100,101]. During infection, pathogens can actively disrupt the mucus barrier, reach the epithelial cell surface, and subsequently create an opportunistic environment for commensal microbes. In response to infection, there are alterations in goblet cell and mucin responses including goblet cell hyperplasia, increased (mucin) secretion, and changes in mucin glycosylation. These changes, in addition to other components of the host immune response, will help to clear the infection. Infection-induced goblet cell hyperplasia and subsequent mucus production may occur by: (1) mediation through antigen presenting cells and lymphocytes. It has previously been shown that T cell derived cytokines play an important role in regulation of goblet cell responses to nematode infection [102]. Thus, immune cells, such as T cells, may be important in infection-induced intestinal goblet cell hyperplasia and subsequent mucus production; (2) directly sampling and recognizing bacteria/pathogens through pattern-recognition receptors (PRRs). Epithelial cells express PRRs such as TLRs and NLRs [74]. Given the close proximity of these cells to commensal and pathogenic bacteria, epithelial and dendritic cells may sample and recognize bacteria through PRRs and generate host immune responses; (3) An emerging concept is the interaction between commensal bacteria and goblet cell response and mucin production. Future studies should investigate whether enteric pathogens can modulate goblet cell biology and mucin production through interaction with host microbiota. Probiotics have been shown to induce Muc2 and Muc3 mucins and inhibit the adherence of enteropathogenic *E.* *coli* suggesting that they may confer protection against infection through alterations in mucin production [77]. The mechanisms underlying the interaction between intestinal microflora and parasites and the role of this interaction in generation of host immune responses remains to be determined.
